# Comparison of gas chromatographic techniques for the analysis of iodinated derivatives of aromatic amines

**DOI:** 10.1007/s00216-023-04713-8

**Published:** 2023-05-20

**Authors:** Nerea Lorenzo-Parodi, Erich Leitner, Torsten C. Schmidt

**Affiliations:** 1 Instrumental Analytical Chemistry, University of Duisburg-Essen, Universitätsstrasse 5, 45141 Essen, Germany; 2 Institute of Analytical Chemistry and Food Chemistry, Graz University of Technology, Stremayrgasse 9/II 8010, Graz, Austria; 3 Centre for Water and Environmental Research, University of Duisburg-Essen, Universitätsstrasse 5, 45141 Essen, Germany; 4 IWW Water Centre, Moritzstrasse 26, 45476 Mülheim an Der Ruhr, Germany

**Keywords:** Gas chromatography-mass spectrometry (GC–MS), Gas chromatography-tandem mass spectrometry (GC–MS/MS), Negative chemical ionization (NCI), Aromatic amines, Derivatization, Urine

## Abstract

Some aromatic amines (AA) have been classified as carcinogens to humans. After entering the body, mainly through tobacco smoke, they can be detected in urine. Thus, their trace analysis as biomarkers in biofluids is of high relevance and can be achieved with gas chromatography (GC–MS), usually after derivatization. This study compares three gas chromatographic methods for the analysis of ten iodinated derivatives of AA: GC–MS in single-ion monitoring (SIM) mode with (1) electron ionization (GC-EI-MS) and (2) negative chemical ionization (GC-NCI-MS), and (3) GC-EI-MS/MS in multiple reaction monitoring (MRM) mode using electron ionization. All methods and most analytes showed good coefficients of determination (*R*^2^ > 0.99) for broad linear ranges covering three to five orders of magnitude in the picogram-per-liter to nanogram-per-liter range, with one and two exceptions for (1) and (2) respectively. Excellent limits of detection (LODs) of 9–50, 3.0–7.3, and 0.9–3.9 pg/L were observed for (1), (2), and (3) respectively, and good precision was achieved (intra-day repeatability < 15% and inter-day repeatability < 20% for most techniques and concentration levels). On average, recoveries between 80 and 104% were observed for all techniques. Urine samples of smokers and non-smokers were successfully analyzed, and p-toluidine and 2-chloroaniline could be found at significantly (*α* = 0.05) higher concentrations among smokers.

**Graphical Abstract:**

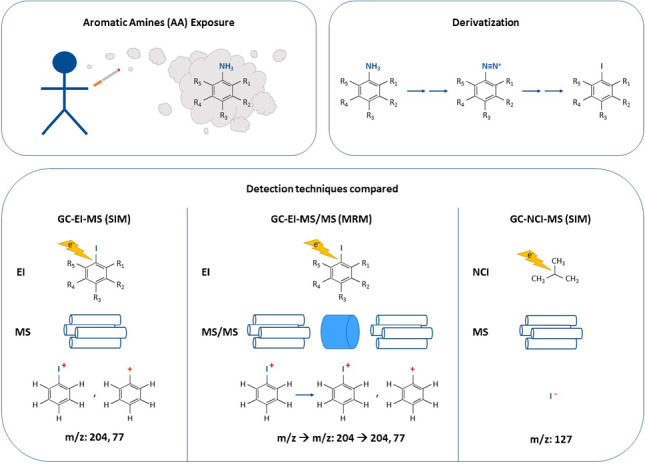

**Supplementary Information:**

The online version contains supplementary material available at 10.1007/s00216-023-04713-8.

## Introduction

Several aromatic amines (AA) have been classified as possible, probable, or certain carcinogens by the International Agency for Research on Cancer (IARC) [1], and most, if not all AA, are believed to have carcinogenic potential [2]. However, they are still widely used, for example, for the production of pharmaceuticals, pesticides, dyes, or rubber [1]. Unfortunately, not only the workers in these industries can get in contact with these substances, but also the general public is at risk: the main source of exposure to some aromatic amines, such as the carcinogenic 2-naphthylamine and ortho-toluidine, and the probable carcinogenic aniline and 4-chloro-o-toluidine, is cigarette smoke [2].

AA enter the blood during the smoking process and are transported into the liver, where they can be metabolized and further transported, for example, to the bladder, where they can react with DNA and proteins to form adducts that can lead to cancer, and can be excreted in the urine [3]. AA have been suggested as the main cause for the excess risk of bladder cancer in smokers [4].

The concentrations of AA in different matrices have been studied in several steps of the aforementioned process, for example, in smoke [5–9] (e.g., aniline, toluidines, or dimethylanilines), as DNA [10–13] and protein adducts [12, 14–17] in cells/blood (e.g., 4-aminobiphenyl), or as free AA and metabolites in urine [3, 18–31] (e.g., naphthylamines, chloroanilines). Because the intake of substances during smoking varies depending on the individual smoking topography [32], and the amount of DNA and protein adducts is typically extremely small [33], this study focuses on urine samples. There, not only free aromatic amines can be found but also their metabolites, such as N-acetylaryl-amine, N-glucuronide arylamine, or hemoglobin and DNA adducts, which can be hydrolyzed and converted back to the free aromatic amines [14, 34].

Direct analysis of AA is possible using liquid chromatography (LC) [19, 21, 24, 25, 35–37]. However, its low peak capacity [38, 39] hinders its use for the analysis of complex urine samples. Due to its high sensitivity, short analysis time, and high resolving power [40], gas chromatography (GC) was used for this study.

In order to reduce the polarity of the AA and facilitate their analysis, they are typically derivatized. In this study, they were iodinated via a Sandmeyer-like reaction as reported by [18, 22, 39]. This derivatization procedure offers the advantage that the reagents used do not need strictly anhydrous conditions, as is the case for the commonly used acylation [39] and silylation derivatizations [40].

This derivatization step enables their analysis with different types of GC systems, such as GC–MS [18, 27, 30, 31, 41, 42], GC × GC–MS [22], GC-NCI-MS [8, 29, 43–45], or GC–MS/MS [3, 9]. However, a comparison of the different techniques, namely GC–MS, GC-NCI-MS, and GC–MS/MS, has not been previously reported for these analytes.

The aim of this study is, therefore, the comparison of different GC detection techniques for the determination of aromatic amines in urine after derivatization to the corresponding iodinated benzenes, namely GC-EI-MS, GC-NCI-MS, and GC-EI-MS/MS. To that end, all studied methods were validated and used for the analysis of real urine samples from smokers and non-smokers.

## Materials and methods

### Chemicals and reagents

Methanol ≥ 99.9%, HiPerSolv Chromanorm for LC–MS (VWR International GmbH, Darmstadt, Germany), was used for the preparation of standard solutions. Iodinated aromatic compounds (Table 1), with a purity of 97% or more, were purchased from Merck KGaA (Darmstadt, Germany).

**Table 1:** Iodinated compounds used, including the abbreviation by which they are referred to in the text, their corresponding aromatic amine precursor, CAS Number (CAS Nr), and purity

Analyte	Abbreviation	Aromatic amine precursor	CAS Nr	Purity (%)
4-Iodotoluene	4IMB	p-Toluidine	624–31-7	99
Iodopentafluorobenzene	IPFB	Pentafluoroaniline	827–15-6	99
2-Iodo-1,3-dimethylbenzene	2I13DMB	2,6-Dimethylaniline	608–28-6	97
Iodobenzene	IB	Aniline	591–50-4	98
1-Chloro-2-iodobenzene	1C2IB	2-Chloroaniline	615–41-8	99
3-Chloro-4-fluoroiodobenzene	3C4FIB	3-Chloro-4-fluoroaniline	156150–67-3	98
2,4,5-Trichloroiodobenzene	245TCIB	2,4,5-Trichloroaniline	7145–82-6	98
2,4-Dichloroiodobenzene	24DCIB	2,4-Dichloroaniline	29898–32-6	98
1-Bromo-4-iodobenzene	1B4IB	4-Bromoaniline	589–87-7	98
2,4-Difluoroiodobenzene	24DFIB	2,4-Difluoroaniline	2265–93-2	98

Concentrated hydrochloric acid (HCl, 37%) from VWR; ethyl acetate (99.9%) from Carl Roth (Karlsruhe, Germany); diethyl ether (99.5%) from ChemLab (Zedelgem, Belgium); sodium hydroxide (NaOH, 99%), alizarin red S (98%), hydriodic acid (unstabilized, 55%), and sodium nitrite (99%) from Merck KGaA; and sodium sulfite (≥ 98%) and sulfamic acid (≥ 99%) from Fluka (Buchs, Switzerland) were used.

### Preparation of stock and standard solutions

All the stock and intermediate solutions were prepared in methanol. Individual stock solutions of each of the analytes were prepared at 1 g/L. An intermediate standard solution was prepared at 1 mg/L for the iodinated aromatic compounds. Working solutions were prepared by diluting the intermediate standard solutions in methanol and were used within 1 month. One working solution was prepared for each concentration tested. All the solutions were stored at 7 °C.

### Sample preparation

Glass-covered stirring bars (VWR International GmbH) were placed in 20-mL crimp vials, which were then filled with 5 mL of the samples and closed with magnetic caps with Silicone/PTFE septa.

For the validation experiments, the samples were prepared by adding 10 µL of the corresponding iodinated working solution to 5 mL deionized water.

Urine samples from seven donors (four smokers and three non-smokers) were collected in 1-L Schott bottles and stored at 7 °C for up to 1 month. The analytes are likely stable under those conditions based on a recent study by Mazumder et al. [46], who found no marked decrease in concentration with samples at similar temperatures during the total time studied, which, however, was limited to 10 days. The urine samples were prepared according to Lamani et al. [22], with a few modifications. First, 20 mL of urine was hydrolyzed with 10 mL of HCl (37%) at 80 °C and 200 rpm stirring speed, for 12 h in order to convert metabolized AA into free AA. All heating and stirring steps were done on an MR 3001 K stirring plate from Heidolph Instruments GmbH & Co. KG (Schwabach, Germany). Once the sample reached room temperature, it was basified by adding 20 mL of 10 M NaOH to the solution. Afterward, the amines were extracted two times into 5 mL of diethyl ether. The organic fractions were then mixed and cleaned with 2 mL of 0.1 M NaOH. The amines were subsequently back-extracted into 10 mL of water, previously acidified with 200 μL concentrated HCl (37%). Any remaining diethyl ether in the aqueous fraction was evaporated by nitrogen blowing on the samples for 20 min.

The aqueous extracts were then derivatized by substituting the nitrogen for an iodine atom in order to decrease the polarity of the extracted amines (see Supplementary information (SI), Fig. S1). This was achieved by adding 200 μL hydriodic acid (55%) and 400 μL sodium nitrite (50 g/L) and stirring at 200 rpm for 20 min (step 1), adding 1 mL of sulfamic acid (50 g/L) and stirring at 200 rpm for another 45 min (step 2), then heating the sample to 95 °C for 5 min (step 3), and finally adding 800 μL sodium sulfite (120 g/L) (step 4) and 200 μL of alizarin red S (1% w/v) (step 5), and adjusting the pH to 5 with NaOH and HCl solutions (step 6). This way, (step 1) the aromatic amines are diazotized and the diazonium ions are further substituted by iodine; (step 2) the surplus of nitrite is destroyed; (step 3) the unreacted diazonium ions are transformed into phenols, and the excess sulfamic acid is destroyed; (step 4) the iodine residue is reduced; (step 5) a pH indicator is added for easy identification of the correct pH; and (step 6) a pH value suitable for subsequent SPME is achieved.

Finally, 5 mL was transferred into a 20-mL vial with a stirrer, crimped, and then placed in the autosampler for further treatment, namely SPME and injection into the GC. For GC-NCI-MS and GC-EI-MS/MS, the samples were diluted 1:10.

### SPME extraction

All the SPME fibers were conditioned prior to their first use, as recommended by the supplier (i.e., 250 °C for 30 min). The SPME extraction was done automatically by different autosamplers, namely HTX PAL (CTC Analytics, Zwingen, Switzerland) for the GC-EI-MS measurements, AOC-6000 (Shimadzu, Kyoto, Japan) for the measurements with the GC-EI-MS/MS, and AOC-5000 Plus for GC-NCI-MS (Shimadzu). All the autosamplers were controlled with PAL Cycle Composer except for the autosampler used in combination with the GC-EI-MS/MS, which was directly controlled by the GCMS Real Time Analysis software (Shimadzu). The samples were incubated for 5 min at 60 °C and 500 rpm in a single magnet mixer (SMM). Afterward, they were extracted from the headspace with a 65-μm PDMS/DVB SPME fiber (1 cm length, Stableflex, 23 Ga, Merck KGaA) for 30 min before injection into the GC system. The SPME fiber remained at least for 5 min in the injector in order to condition the fiber after injection, except for the GC-EI-MS/MS measurements, where the fiber was pre-conditioned for 2 min in a dedicated conditioning station at 280 °C, and remained for 2 min in the injector.

The extraction efficiencies of the three SPME fibers used (one for each technique) were compared after the methods were validated, using GC-NCI-MS and a iodinated derivatives solution (1 ng/L, Fig. S2 and SI). Furthermore, a SPME test mix (200 ng/L) was analyzed regularly in order to ensure the integrity of the fiber and the performance of the system, by adding 20 µL of a stock solution in a vial with a stirrer. A list of the analytes included in the mix (minimum purity 95%, different providers) can be seen in Table S1 (SI). Because the mix included mostly analytes that are not ionizable by GC-NCI-MS, it was not used for this technique.

A significantly lower intensity was observed with GC-EI-MS/MS during the first use of the corresponding SPME fiber (see Fig. S3, SI). Although the manufacturer instructions were followed, it has been previously reported that it might be insufficient conditioning [47, 48]. Therefore, the GC-EI-MS/MS results were normalized according to the SPME Mix intensities, which, as expected, also showed a similar trend (see Table S1, SI).

### GC–MS analysis

In order to facilitate the comparison of the different techniques, as many parameters as possible were kept constant throughout the different devices. Helium 5.0 (Linde, Höllriegelskreuth, Germany) was used as carrier gas for all techniques.

A GCMS-QP2010 Ultra (Shimadzu) equipped with a ZB-Wax 20 m × 0.18 mm × 0.18 µm (Phenomenex, CA, USA) was used for the GC-EI-MS analysis. The linear velocity was set to 45 cm/s, which corresponds to a column flow of 1.03 mL/min. The samples were injected in splitless mode, and after a sampling time of 1 min, the split ratio was set to 10. The injector temperature was set to 250 °C, the interface temperature to 230 °C, and the ion source temperature to 200 °C. The oven program started at a temperature of 40 °C, was held for 1 min, ramped at a rate of 10 °C/min to 240 °C, and held for 1 min. The final oven temperature was lower than in other instruments due to the different column used. The acquisition was made in SIM mode, with an event time of 0.2 s. Twenty channels were looked into (Table S2 (SI)), typically corresponding to the molecular ions and the fragment resulting from the loss of iodine.

A GCMS-TQ8050 (Shimadzu) with a 30 m × 0.25 mm × 0.25 µm Rxi-5MS (Restek, PA, USA) was used for the GC-EI-MS/MS analysis. The injector, interface, and ion source temperatures were set to 270, 280, and 200 °C respectively. The injection was done in splitless mode, and a split ratio of 10 was applied after a sampling time of 1 min. The linear velocity was 35 cm/s. The oven starting temperature was 40 °C, which was held for 1 min, ramped to 280 °C at a rate of 10 °C/min, and held for 1 min. The MS was operated in multiple reaction monitoring (MRM) mode, using argon 5.0 (Linde) as the collision gas. The optimal collision energies (CE) were found by directly injecting 1 µL of a mixture of the iodinated analytes in 50:50 methanol:ethyl acetate (0.5 mg/L), at different CE, and comparing the intensities. The transitions monitored, and their corresponding CE, can be seen in Table S3 (SI). The event time was set to 0.3 s for all transitions.

GC-NCI-MS measurements were done on a GCMS-QP2010 Plus system (Shimadzu) equipped with a 30 m × 0.25 mm × 0.25 µm Rxi-5Sil MS column (Restek). The interface temperature was set to 250 °C and the ion source temperature to 160 °C. All other GC parameters were the same as for the GC–MS/MS. MS acquisition was performed in SIM mode, with an event time of 0.3 s, and monitoring the ions corresponding to chlorine (35, 37), bromine (79, 81), and iodine (127). The ionization gas was isobutane 3.5 (Linde), set to a pressure of 0.7 bar.

A comparison of the chromatograms obtained with the aforementioned parameters can be seen in Fig. S4 (SI).

### Method validation

The validation was done mostly according to the Eurachem Guide [49]. The raw data were evaluated with GCMS﻿solution (Shimadzu) without applying smoothing, and the calculations were performed in Excel (Microsoft).

First of all, the linear ranges were studied with the aim of seeing not only how sensitive the instruments can be, but also whether they have a linear response at the concentration levels expected for real samples. Therefore, a very broad range was studied, and, subsequently, a logarithmic scale was used in order to have equidistant calibration levels, as recommended by the DIN 38402–51 [50]. Concentrations from 1 pg/L to 500 ng/L were tested, with three concentration levels per order of magnitude. Exemplary calibration curves for each technique can be seen in Fig. S5 (SI).

Afterward, the limits of detection (LODs) and of quantification (LOQs) were studied by repeating ten times the analysis of a calibration level where most of the analytes showed a signal to noise ratio (S/N) between 6 and 15 (200 pg/L for GC-EI-MS, 100 pg/L for GC-NCI-MS, and 10 pg/L for GC-EI-MS/MS). This was done so that the concentrations used for the calculation of the limits were not extremely high in comparison with the limits themselves, and to consequently avoid obtaining overestimated sensitivities. Because the calibration curves were up to five orders of magnitude (1–100,000 pg/L) broad, and the points were equidistant only in the logarithmic scale, a normal linear fit would be very heavily influenced by the higher calibration levels. Therefore, in order to accurately determine lower concentrations, we limited the number of calibration levels in this and the following sections, so that there would be at least 5 points per curve and up to 7 levels. Furthermore, the concentration to be determined was, if possible, kept in the middle of the levels selected. Afterward, the limits were calculated according to the Eurachem Guide [49], with a constant (*k*, equal to 3 for LODs and 10 for LOQs) multiplied by the standard deviation of the replicate concentrations and divided by the degrees of freedom (*n* − 1 = 9).

The next step was to calculate the intra-day and inter-day repeatability. In order to do that, at least three calibration points (equidistant in the logarithmic scale and well distributed within the linear range) were measured in triplicate over three consecutive days. The concentration levels tested were 1, 10, and 100 ng/L for GC-EI-MS; 0.1, 1, and 10 ng/L for GC-NCI-MS, and, because of the broad range that could be analyzed with GC-EI-MS/MS, four concentrations, namely 0.01, 0.1, 1, and 10 ng/L, were studied with that technique.

The recovery was calculated from the repeatability experiments. For each instrument and concentration level, the recovery was calculated by dividing the average of the concentrations obtained (*n* = 9) by the expected theoretical concentration and multiplying the result by 100.

## Results and discussion

Ten aromatic amine derivatives (i.e., iodinated aromatic compounds) were measured directly, without further sample treatment, in order to facilitate the direct comparison of the methods. The studied analytes were selected as model compounds due to their diverse chemical structures and properties. Furthermore, most of them have been previously studied and found in smoke [5–9], blood/tissue [10, 14–16], and/or urine matrixes [3, 21–23, 26, 28–30]. Three of the most comprehensive papers in terms of AA studied [8, 22, 28] found aniline, p-toluidine, 2,6-dimethylaniline, 2-chloroaniline, 2,4,5-trichloroaniline, and 2,4-dichloroaniline, which are also included in this research.

### Linear range

The linear ranges observed can be found in Table 2. For the GC-NCI-MS experiments, a plateau in the linear curve could be observed at concentrations of 50 or 100 ng/L, depending on the compound. Excellent goodness of fit was achieved for all the methods tested, with coefficients of determination (*R*^2^) above 0.99 for all cases except for 1B4IB and 245TCIB when measured with GC-NCI-MS (0.988 and 0.989 respectively, data not shown).

**Table 2:** Limits of detection (LOD), quantification (LOQ), and linear ranges in picograms per liter, obtained for the iodinated, aromatic compounds with the studied GC methods. The concentration ranges tested were 20–500,000 pg/L for GC-EI-MS, 2–100,000 pg/L for GC-NCI-MS, and 1–100,000 pg/L for GC-EI-MS/MS. LODs and LOQs were calculated with concentrations where most analytes had S/N between 6 and 15, namely 200 pg/L for GC-EI-MS, 100 pg/L for GC-NCI-MS, and 10 pg/L for GC-EI-MS/MS

	GC-EI-MS			GC-NCI-MS			GC-EI-MS/MS		
	LOD (pg/L)	LOQ (pg/L)	Linear range (pg/L)	LOD* (pg/L)	LOQ* (pg/L)	Linear range (pg/L)	LOD (pg/L)	LOQ (pg/L)	Linear range (pg/L)
IPFB	30	99	100–500,000	7.3	19	50–50,000	0.9	2.9	5–100,000
24DFIB	50	167	200–500,000	4.7	31	10–20,000	0.9	2.9	2–100,000
IB	25	84	100–500,000	6.8	23	20–50,000	2.1	7	5–100,000
4IMB	21	71	100–500,000	5.2	31	20–50,000	1.1	3.5	1–100,000
3C4FIB	14	47	100–500,000	6.3	21	10–20,000	1.3	4	2–100,000
1C2IB	26	86	100–500,000	3.0	15	5–20,000	0.8	2.5	2–100,000
2I13 DMB	21	71	50–500,000	5.6	28	10–50,000	0.5	1.7	1–100,000
1B4IB	-	-	10,000–500,000	4.9	14	20–50,000	2.0	7	10–100,000
24DCIB	9*	29*	50–500,000	4.8	20	10–20,000	1.2	4.0	2–100,000
245 TCIB	28	93	200–500,000	6.3	13	10–50,000	3.9	13	10–100,000

*Outliers found with Dixon’s *Q* test (*α* = 0.05, *Q*_Critical_ = 0.412) not included in the calculations

LODs and LOQs were calculated according to the Eurachem Guide [49], as a constant (3 and 10, respectively) multiplied by the standard deviation of the concentration from tenfold replicates, and divided by the degrees of freedom (*n* − 1 = 9). Smoothing was set to “none”

The determination of 1B4IB with the GC-EI-MS method was hindered by an interfering signal covering the peak (see Fig. S6, SI), which led to the analyte being identified only in concentration levels of 10 ng/L or above. A different column was used with this instrument, which could have led to a different elution pattern and may explain why the interference was not observed in the other systems. A different set of m/z may be used to study this compound, such as 155 and 157, which corresponds to the fragment without iodine.

When compared with literature (Table 3), the results are similar or better than those typically reported. In most cases, a linear range of approximately 3 orders of magnitude is reported [20–24, 27, 29, 30]. The results presented here show a linear range of 4 orders of magnitude for GC-EI-MS and GC-NCI-MS and of 5 for GC-EI-MS/MS. The broader the linear range, the higher the likelihood that analytes at very low concentrations can be accurately quantified, and that there is no further dilution needed for samples with very high concentrations, saving both sample volume and time.

**Table 3:** Figures of merit of most recent literature regarding the analysis of aromatic amines from urine samples. Ranges reported correspond to the minimum and maximum values from different analytes and/or concentration levels. Data in parentheses indicate missing experimental information needed for its interpretation

Der. reagent	Injection technique	Volume/SPME fiber	Instrument	Calibration range (ng/L)	LOD (ng/L)	Recovery (%)	Intra-day precision (%)	Inter-day precision (%)	Concentration in real samples	Ref
HI	HS-SPME	110 μm PDMS/DVB	GC–MS	100–1200	3–12^2^	n.r	3–12	n.r	NS: n.d., S: n.d.–243 ng/L	[18]
No	LI	5 µL	LC–MS/MS (MRM)	100–50,000	25–500^2^	75–114^a^	1.6–11.7	2.1–15.9	U: n.d.–1.5, S: n.d.–3.47 µg/L	[19]
TMA-HCl, PFPA	LI	1 µL	GC–MS/MS (EI, MRM)	482–1280	1.8–111.2^1^	> 85	1.1–6.3	2.6–6.3	n.r	[3]
No	HS-SPME	80 µm, JUC-Z2	GC–MS/MS (MRM)	50–100,000	(0.010–0.012)^2^	95–101	(7.1–7.7)	n.r	NS: n.d., S: 68.4–123.1 ng/L	[20]
PFPA, Pyr	LI	5 µL	LC–MS (SIM)	1000–1,000,000	1000^3^	n.r	n.r	n.r	NS: 1–1.13, S: 1.46–2.33 µg/L	[21]
HI	HS-SPME	65 μm PDMS/DVB	GC × GC–MS	1–500	5.2–24.4^4^	n.r	n.r	n.r	n.r	[22]
No	LI	1 µL	GC-FID	30–100,000	(7–10)^2^	93.0–99.9	2.5–5.9	4.7–7.3	NS: n.d.–1.2, S: 2–14.5 µg/L	[23]
No	LI	10 µL	LC–MS/MS (MRM)	5–10,000	(1.5–5)^5^	88–111	6.1–8.9	9.0–9.9	NS: 1.11–12.32, S: 5.39–67.02 ng/24 h	[24]
No	LI	3 µL	UFLC (UV–Vis)	5000–500,000	(DI: 39,600–94,400, AE: 880–1300)^2^	89–105	0.6–7.9	2.4–10	U: n.d.–12.8 µg/L	[25]
No	HS-SPME	PEG/CNTs	GC-FID	1–105	(0.5–50)^2^	63.7–97.0	3.2–9.1	5.5–12.0^a^	NS: n.d.–940, S: 1140–50,960 ng/L	[26]
TMA-HCl, PFPA	LI	1 µL	GC–MS/MS (EI, MRM)	50–25,000	0.89^6^	(20–25)	1.7–6.7	7.5–8.4	NS: 1.30–2.07, S: 7.43–10.16 pg/mg Cr	[27]
No	LI	1 µL	GC–MS (SIM)	5–60,000	2–26^7^	94–104	4.5–6.2	6.0–6.8	U: n.d.–690 ng/L	[28]
PFPA, Pyr	LI	0.2 µL	GC–MS (NCI, SIM)	10–2500	(1–4)^2^	94–107	(2.7–4.6)	(5.1–7.0)	NS: 9.6–105.2, S: 15.3–204.2 ng/24 h	[29]
PFPA, Pyr	LI	1 µL	GC–MS (SIM)	100–100,000	(50–2000)^2^	70–125	1.8–14	7.5–19	U: n.d.–3.5 µg/L	[30]
PFPI	LI	1 µL	GC–MS (SIM)	n.r	(0.05 ng)^3^	(82.3–96.8)	n.r	n.r	NS: n.d.–1073.4, S: 3.6–2119.8 ng/24 h	[31]
HI	HS-SPME	65 μm PDMS/DVB	GC-EI-MS	0.05–500	0.009–0.05^8^	93–116	2.8–11	1.8–46	NS: n.d.–64, S: n.d.–173 ng/L	This study
			GC-NCI-MS	0.005–50	0.003–0.007^8^	71–104	2.1–12	3.7–40		
			GC-EI-MS/MS	0.001–100	0.001–0.004^8^	66–117	0.2–13^c^	4.0–35		

Abbreviations: *AE* after extraction, *Cr* creatinine, *Der*. derivatization, *DI* direct injection, *HS-SPME* headspace solid-phase microextraction, *JUC-Z2* two-dimensional porous organic framework, *LI* liquid injection, *LOD* limit of detection, *n.d.* not detected, *n.r.* not reported, *NS* non-smoker, *PDMS/DVB* polydimethylsiloxane/divinylbenzene, *PEG/CNTs* poly(ethylene glycol) modified with multi-walled carbon nanotubes, *PFPA* pentafluoropropionic anhydride, *PFPI* pentafluoropropionyl-imidazol, *Pyr* pyridine, *Ref* reference, *S* smoker, *TMA-HCl* trimethylamine hydrochloride, *U* unknown smoking status, *UFLC* ultra-fast liquid-chromatography

^a^Considering 37 of the 41 analytes studied.

^b^Batch-to-batch precision.

^c^Excluding 24TCIB (28%) and IB (19%) at a concentration level of 10 pg/L

LOD calculations:

^1^according to CLSI EP17-A

^2^S/N (signal to noise ratio) = 3

^3^not reported

^4^according to DIN 32645

^5^according to FDA guideline

^6^SD_low-quality-control samples_

^7^LOD = 3*SD_*y*/*x*_/*b* (_*y*/*x*_ = regression)

^8^LOD = 3*SD/(*n* − 1), (*n* = degrees of freedom), according to Eurachem Guide [49]

### LODs and LOQs

As expected, GC-EI-MS/MS shows the lowest LODs and LOQs, followed by GC-NCI-MS and GC-EI-MS (see Table 2), which on average have 3 and 12 times higher LODs, respectively. The high sensitivity achieved with GC-NCI-MS can be attributed to the high selectivity of this technique for halogenated compounds, which have a high electron affinity. The even better results obtained with GC-EI-MS/MS can be explained by the fact that in the first quadrupole, only the ions selected are trapped, which decreases the background noise, and consequently increases the sensitivity significantly.

LODs and LOQs for the analysis of aromatic amines in urine are generally reported in the nanogram-per-liter range (see Table 3). The best LODs reported (< 5 ng/L) were achieved with MS/MS detectors [20, 24, 27] and GC-NCI-MS [29] systems, while the worst (> 50 ng/L) were observed with EI-MS detectors [21, 30]. A similar trend can be observed in this study (Table 2), where GC-EI-MS shows worse limits than the other methods tested. Nonetheless, the results obtained were better than most of those found in literature, with LODs of 9–50 pg/L for GC-EI-MS, 3.0–7.3 pg/L for GC-NCI-MS, and 0.5–3.9 pg/L for GC-EI-MS/MS. The lowest limit reliably reported for aromatic amines in urine is 0.89 ng/L [27], which is between 120 and 1800 times worse than those reported here for the iodinated derivatives with GC-NCI-MS and GC-EI-MS/MS. The reason for the higher sensitivity achieved here is most likely the combination of a pre-concentration step like SPME with very sensitive measurement techniques and the fact that iodinated derivatives were measured directly. Taking into account that during a similar derivatization procedure, for most analytes an estimated loss of 10% was observed [38], it would be expected that the limits found with these instruments, including the complete sample preparation, would still be comparable if not better than those found in literature.

Other factors can affect the sensitivity of the method, such as the amount of sample used (typically within 5–20 mL), the concentration level studied, the steps of the sample preparation procedure included, the use of matrix-matched calibrations, and the equations used for the calculations (signal to noise ratio, standard deviation, etc.). Unfortunately, in several occasions, information was lacking for a proper interpretation of the results. For example, when the limits were calculated based on the S/N ratio, the concentrations used or if smoothing was applied was usually not reported. If too-high concentrations are used, this can lead to too-low LODs, which seems to be the case for the lowest limit found in literature [20], where the extrapolated limit reported is more than three orders of magnitude lower than the linear range.

### Precision: intra-day and inter-day repeatability

Intra-day repeatability (reported as relative standard deviations or RSDs) values were on average below 15% for all analytes, concentration levels, and measuring techniques (Table 4). These results are in agreement with literature, as seen in Table 3, despite the fact that generally lower concentrations are used in this study, and a decrease in precision can be expected at lower concentration levels.

**Table 4:** Average intra-day and inter-day repeatability, and recovery (%) results obtained for each of the techniques studied. The concentration levels tested were as follows: for GC-EI-MS, L (low) = 1 ng/L, M (medium) = 10 ng/L, and H (high) = 100 ng/L; for GC-NCI-MS, L = 0.1 ng/L, M = 1 ng/L, and H = 10 ng/L; and for GC-EI-MS/MS, L = 0.01 ng/L, M-L = 0.1 ng/L, M-H = 1 ng/L, and H = 10 ng/L

	GC-EI-MS			GC-NCI-MS			GC-EI-MS/MS			
	L	M	H	L	M	H	L	M-L	M-H	H
Intra-day repeatability (%, *n* = 9)	7.1	7.8	5.2	5.6	5.5	3.7	12	4.0	2.8	2.1
Intra-day repeatability (%, *n* = 9)*	3.8	5.7	2.0	3.9	2.9	1.6	10.3	3.5	1.5	1.0
Inter-day repeatability (%, *n* = 3)	25	15	7.7	13	27	24	15	16	21	15
Inter-day repeatability (%, *n* = 3)*	12.6	8.7	4.5	5.3	7.4	2.9	13.2	9.7	9.6	7.7
Recovery (%, *n* = 9)	102	104	96	83	94	80	92	89	88	80

*Results obtained after internal standard-equivalent correction (explained in SI)

For all three methods, as expected, the repeatability improves with increased concentration. For GC-EI-MS, it is not apparent at first; however, the method is not sensitive enough to detect 1B4IB at the lower concentration, which would significantly worsen the average repeatability of that concentration. If the same concentration level is compared across methods, for example, 1 ng/L, the average intra-day repeatabilities of the methods are 7.1% for GC-EI-MS (without IB4IB), 5.5% for GC-NCI-MS, and 2.8% for GC-EI-MS/MS. The individual intra-day repeatabilities of each analyte can be seen in Table S4, SI.

The majority of the inter-day repeatability results (reported as RSDs) are below 20%; however, there are some exceptions. This could be due to the fact that *n* is smaller (3 vs 9), and the typical errors introduced during sample preparation and measurement have a bigger effect the smaller the number of samples measured.

If the different replicates are studied over time (as exemplified for 10 ng/L in Fig. S7, SI), a clear pattern appears for the GC-NCI-MS results. This decrease over time can be explained by the fact that the ionization gas used (isobutane 3.5) is not as pure as the gases typically used for gas chromatography (5.0 or above), leading to the ion source becoming dirty with a corresponding decrease of the resulting signals. Unfortunately, to the best of our knowledge there is no purer isobutane commercially available, and the other gases that are typically used present other disadvantages (namely, methane induces harder ionization, and ammonia results in more maintenance needed). Therefore an equivalent to an internal standard correction, based on the averaged response of all the analytes instead of one specific standard, was made (as explained in SI and exemplified in Table S5), and much better precision results (below 15% in all cases) were achieved (Table 4 and Fig. S8, SI). Because of how fast the intensity decreases, GC-NCI-MS would not be recommended, even if an internal standard is used, for larger sample batches.

### Recoveries

On average, recoveries between 80 and 120% were obtained for all techniques and concentration levels studied, with RSDs between 3 and 14% (see Table 4 and Table S6 (SI) for a more detailed table with recoveries for each analyte).

As mentioned in “LODs and LOQs,” it was not possible to always select the calibration curve used so that the concentrations studied were in the middle. This could explain why some recoveries for the lower and higher levels appear to be worse. However, it needs to be kept in mind that up to 4 different levels were tested for recoveries, when typically only one is reported. This was the case because the overall performance of the three instruments was to be compared. In the case of real samples, it is recommended that a smaller calibration curve is used, with more points per order of magnitude.

During the method optimization for GC-EI-MS/MS, the measuring windows were set relatively narrow in order to have better selectivity. However, because during later experiments the intensity of the peaks increased, as explained in “SPME extraction” and the SI, a higher tailing than expected was observed, and, in some cases, cut off due to the window length (see Fig. S9, SI). Therefore, especially for the higher concentration levels, a worse recovery can be observed. This could be avoided by increasing the observed window, or, alternatively, by using a higher split ratio.

The overall recovery range found in the literature is between 64 and 125%, although generally, it is between 80 and 110% (Table 3). Despite the fact that the concentration levels used in the literature are typically higher than in this study, the recoveries observed are in agreement.

### Real samples

GC-NCI-MS and GC-EI-MS/MS show extremely good sensitivities, which allows for the analysis of derivatized AA in the picogram-per-liter range. A few derivatized AA can be often found in higher concentrations, which could lead to some analytes being outside of the calibration curves. If these AA were the main interest, this could be easily solved by diluting the samples before measuring them, which would provide the added advantage of reducing the matrix interference and therefore increasing the robustness of the analysis. This could also be an advantage when measuring archived samples, since instead of diluting after the sample preparation is done, less urine sample could be used to start with. Alternatively, if high- and low-concentration AA need to be analyzed, the GC-EI-MS/MS method could be adjusted by changing the Q1 or Q3 resolutions so that the sensitivity in those highly concentrated compounds is lower compared to those of the rest of the compounds.

In this study, the validated methods were used and the samples were diluted to avoid saturation. In most cases, IB still showed concentrations above the highest calibration point. This analyte is typically found in both smokers and non-smokers in high concentrations, which means there is another source of exposure besides tobacco smoke. Therefore, when analyzing the concentrations of aromatic amines in relation to smoking status, and in order to avoid saturation of the detector in scan methods, this analyte could be left out.

**Fig. 1:**
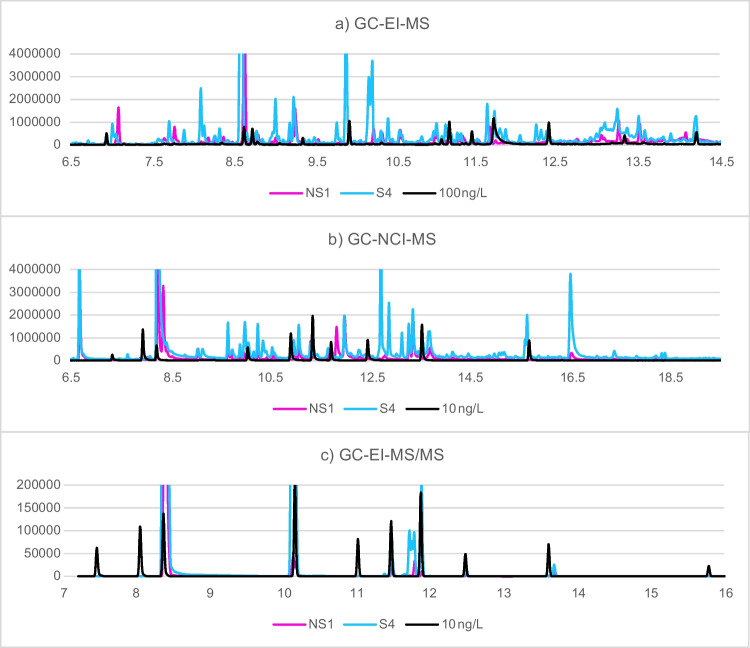
Chromatogram comparison of pink = NS1, blue = S4, and black = 100 ng/L for **a** GC-EI-MS or 10 ng/L for **b** GC-NCI-MS, and **c** GC-EI-MS/MS, zoomed. The m/z shown are **a** the quantifier and qualifier ions reported in Table S2 (SI), **b** 127 and **c** the transitions reported in Table S3 (SI). NS1 and S4 were diluted 1:10 for **b** and **c**

With all three techniques, more aromatic amines could be tentatively identified in the samples from smokers than non-smokers (see Table 5). As expected, with GC-NCI-MS, the most aromatic amines could be tentatively identified. This is due to the fact that with this technique, m/z = 127 was one of the monitored ions. This ion corresponds to the loss of iodine and, due to the derivatization process, is to be expected in all the aromatic amines in the sample. Because the GC-EI-MS analysis was done in SIM mode, only those compounds with the studied m/z (Table S2, SI) could be detected. This technique is the least specific, as also non-aromatic compounds are detected, and therefore have to be filtered out manually. Finally, GC-EI-MS/MS in MRM mode is the most selective technique, and the best option among those tested for target screening, as it only detects molecules with defined transitions within defined measuring windows. Nonetheless, a few isomers could still be detected. An exemplarily chromatogram from a smoker’s and non-smoker’s sample can be seen in Fig. 1.

**Table 5:** Total number of tentatively identified derivatized aromatic amines with each technique, in urine samples from three NS = non-smokers and four S = smokers. All peaks found were taken into account for the GC-NCI-MS and GC-EI-MS/MS techniques, and only peaks with a loss of 127 were included in the GC-EI-MS calculations

	NS1	NS2	NS3	S1	S2	S3	S4
GC-EI-MS	37	39	38	41	41	42	45
GC-NCI-MS	55	55	49	61	79	74	68
GC-EI-MS/MS	13	15	14	16	16	16	16

Six of the analytes studied could also be quantified with at least two techniques in most samples (Table 6). Great variability could be observed, as expected due to the nature of the samples, which could be partially accounted for by normalizing to creatinine and thereby correcting urinary output differences. Nonetheless, the averaged concentrations in samples from smokers were higher than in samples from non-smokers for all six analytes. A similar trend can be observed in literature (Table 3). Furthermore, 4IMB and 1C2IB were found at significantly higher concentrations in smokers’ samples, as determined by either Welch’s two-sided *t*-test or the two-variable *t*-test (*α* = 0.05) [51, 52], and thus may be good candidates for future biomarker studies.

**Table 6:** Calculated concentrations in urine samples from three NS = non-smokers and four S = smokers, in nanograms per liter, based on the average of the three techniques studied. Average NS and S concentrations are presented in bold

	NS1	NS2	NS3	NS	S1	S2	S3	S4	S
4IMB	31	64	25	**40**	78	173	130	145	**132**
3C4FIB	0.6	0.6	0.5	**0.6**	0.6	0.4	1.0	0.9	**0.8**
1C2IB	13	17	13	**15**	22	19	34	29	**26**
2I13DMB	2.5	3.9	1.6	**3**	5.2	24	21	46	**24**
24DCIB	0.2	0.2	0.3	**0.3**	0.3	0.4	0.5	0.8	**0.5**
245TCIB	0.5	0.7	1.2	**0.8**	2.9	0.8	0.8	0.5	**1.2**

The three techniques show comparable results, most of the time within the same order of magnitude. Despite the extra dilution step, and because of the high sensitivity of the technique, IPFB, 24DFIB, and 1B4IB could only be detected with GC-EI-MS/MS in most samples (Table S7, SI). 1C2IB shows the highest similarities between the three techniques, with RSDs below 20% for all samples. 2,4DCIB could not be detected with GC-NCI-MS but also showed RSDs below 20% for the other two techniques. In several cases, the higher deviation was due to co-elutions present with some of the techniques (see SI). Depending on the analytical requirements, the GC parameters could be optimized to resolve specific co-elutions. Furthermore, the use of internal standards could have a positive effect minimizing the deviations between the techniques.

## Conclusion

The most promising technique for the analysis of the iodinated derivatives of aromatic amines in urine is GC-EI-MS/MS. Despite showing slightly worse recoveries than GC-EI-MS, the obtained results are still within acceptable ranges. Furthermore, as expected, the sensitivity and selectivity of the method are significantly better, so that GC-EI-MS/MS would be the method of choice for further analysis. GC-NCI-MS shows a slightly worse behavior than GC-EI-MS/MS, with the addition of the significant loss in sensitivity over time due to the ionization gas purity. Nonetheless, for qualitative/non-target analysis, GC-NCI-MS offers the advantage that all the derivatized iodinated amines can be easily identified. Finally, GC-EI-MS shows the worst results in terms of sensitivity and selectivity. However, it has the advantage of being the most widespread and least expensive of the three techniques studied. This technique could therefore be especially interesting when low concentrations are not of interest, or for screening purposes.

One of the main drawbacks of GC-EI-MS/MS in MRM mode is that the analytes need to be defined in advance. This could be problematic when measuring real samples, since approximately 150 different AA have previously been identified in smokers’ urine [22]. Most GC-EI-MS/MS offer the possibility of doing scan/MRM, which could enable qualitative non-target screening and the sensitive and selective quantification of specific target compounds. Another alternative would be to combine the derivatization method presented here with GC-EI-MS/MS in neutral loss mode.

Finally, the high sensitivity and selectivity obtained for the analysis of the iodinated derivatives with HS-SPME GC-EI-MS/MS are a great advantage over other methods found in literature. Especially for the analysis of valuable samples, such as archived samples (for example, from cohort studies), since it can enable a considerable reduction of sample volume needed. This could be used to foster our understanding of the interactions between the smoking status, the concentration of aromatic amines, and the risk of developing smoking-related diseases.

## Supplementary Information

Below is the link to the electronic supplementary material.

Supplementary file1 (DOCX 345 kb)
