# Supplementary material for: Comparison of gas chromatographic techniques for the analysis of iodinated derivatives of aromatic amines
Source: Anal Bioanal Chem. 2023 May 20;415(17):3313–25. doi: 10.1007/s00216-023-04713-8 (PMC10289911; doi:10.1007/s00216-023-04713-8)
Supplement: Supplementary file 1 — Supplementary file1 (DOCX 345 kb) [file 216_2023_4713_MOESM1_ESM.docx]

**Supplementary Information**

For the paper **Comparison of gas chromatographic techniques for the analysis of iodinated derivatives of aromatic amines,** by Nerea Lorenzo-Parodi, Erich Leitner, Torsten C. Schmidt.

**Derivatization procedure**


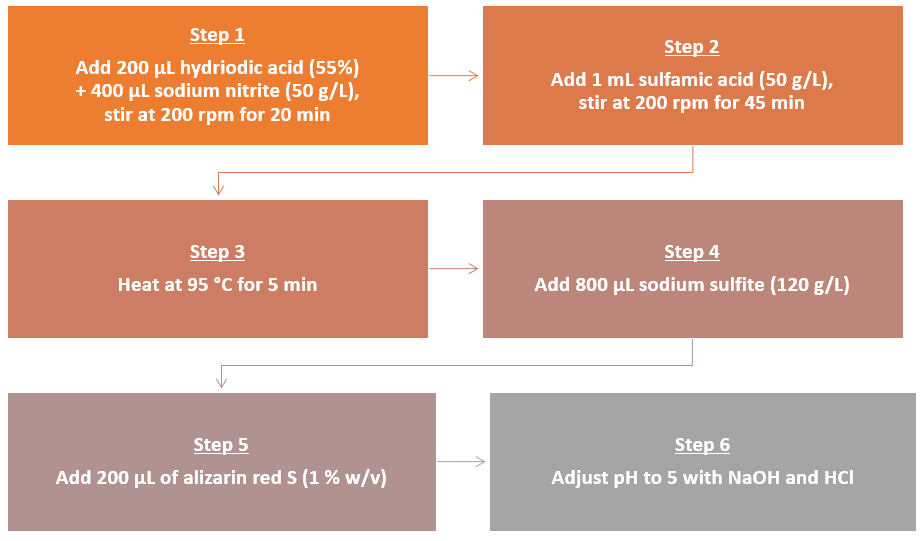


Fig. S 1. Derivatization procedure followed during sample preparation.

**Fiber comparison**

1 ng/L solutions of the iodinated derivatives were measured in triplicate with GC‑NCI‑MS, with each of the fibers used in this study. The results show that the fibers were comparable for most of the analytes, however, for 4IMB, 3C4FIB, and 1B4IB, the fiber used for the GG‑MS/MS measurements showed significantly worse results (tested with one-way ANOVA, and shown in Fig. S 2). This could have an effect on the linear ranges and limits presented for those analytes when measured with GG‑MS/MS. It would be expected that if the other fibers had been used, the results for these three analytes with GC-EI-MS/MS technique would have been even more sensitive.

Fig. S 2. Comparison of the fibers used for each of the three techniques, by measuring 1 ng/L with GC-NCI-MS at the end of the experiments.

**SPME Mix corrections for GC-EI-MS/MS**

The day the calibration curve was measured (08. Feb), the intensities of the SPME Mix were much smaller than for the rest of the days, see Fig. S 3. Which might indicate insufficient conditioning ^[1, 2]^, despite following the manufacturer instructions.

Fig. S 3. Peak areas of a selected number of analytes from the SPME mix over time, measured with GC-EI-MS/MS.

In order to correct for the significantly smaller intensity observed the first day, the results obtained in each of the experiments where GC-EI-MS/MS was used were normalized based on the SPME Mix intensity by correcting each day with the factor shown in Table S 1.

Table S 1. Relative peak areas of the SPME mix analyzed, normalized with the areas of the day the calibration curve was measured (08. Feb), and the correction factors used, which were calculated by normalizing the sum of the relative areas of each day with that of the 08. Feb.

|  | 08. Feb | 10. Feb | 11. Feb | 12. Feb | 13. Feb |
| --- | --- | --- | --- | --- | --- |
| Heptanal | 1.00 | 2.26 | 2.26 | 2.70 | 2.14 |
| α-Pinene | 1.00 | 1.61 | 1.39 | 1.62 | 1.57 |
| β-Pinene | 1.00 | 1.82 | 1.59 | 1.76 | 1.69 |
| Octanal | 1.00 | 2.14 | 2.37 | 2.57 | 2.27 |
| n-Decane | 1.00 | 2.00 | 1.83 | 2.11 | 2.12 |
| p-Cymene | 1.00 | 1.90 | 1.86 | 2.09 | 1.91 |
| 1,8-Cineol | 1.00 | 2.13 | 2.42 | 2.73 | 2.20 |
| Nonanal | 1.00 | 3.87 | 3.86 | 4.34 | 4.06 |
| L-Menthol | 1.00 | 2.74 | 2.46 | 3.36 | 3.09 |
| n-Dodecane | 1.00 | 2.50 | 2.56 | 3.03 | 2.97 |
| cis-Carveol | 1.00 | 2.50 | 1.72 | 2.39 | 2.17 |
| trans-Carveol | 1.00 | 2.63 | 1.84 | 2.44 | 2.29 |
| Carvone | 1.00 | 2.83 | 2.74 | 3.39 | 2.97 |
| 1-Decanol | 1.00 | 3.08 | 2.32 | 3.74 | 3.38 |
| 1-Undecanol | 1.00 | 2.70 | 2.01 | 3.07 | 2.63 |
| n-Tetradecane | 1.00 | 2.50 | 3.08 | 3.22 | 3.10 |
| 1-Dodecanol | 1.00 | 2.80 | 2.03 | 2.87 | 2.34 |
| Sum | 17 | 42.0 | 38.3 | 47.4 | 42.9 |
| Correction factor | 1.00 | 2.47 | 2.26 | 2.79 | 2.52 |

**Mass spectrometric parameters**

Table S 2. Mass spectrometric parameters for the GC-EI-MS analysis, including the quantifier and qualifier ion recorded for each of the analytes studied, and the corresponding monoisotopic masses calculated based on [3].

| **Analyte** | **Monoisotopic mass (Da)** | **Quantifier ion (m/z)** | **Qualifier ion (m/z)** |
| --- | --- | --- | --- |
| IPFB | 293.8965 | 294 | 117 |
| 24DFIB | 239.9248 | 240 | 113 |
| IB | 203.9436 | 204 | 77 |
| 4IMB | 217.9592 | 218 | 91 |
| 3C4FIB | 255.8952 | 256 | 129 |
| 1C2IB | 237.9046 | 238 | 111 |
| 2I13DMB | 231.9749 | 232 | 105 |
| 1B4IB | 281.8541 | 282 | 284* |
| 24DCIB | 271.8657 | 272 | 145 |
| 245TCIB | 305.8267 | 306 | 308* |

Table S 3. Mass spectrometric parameters for the GC-EI‑MS/MS analysis in MRM mode, including start and end recording times, precursor and product ions, and collision energies (CE) for each of the analytes. The event time was set to 0.3 s for all transitions.

| **Analyte** | **Start time (min)** | **End time (min)** | **Precursor ion (m/z)** | **Product ion 1 (m/z)** | **Product ion 2 (m/z)** | **CE 1 (V)** | **CE 2 (V)** |
| --- | --- | --- | --- | --- | --- | --- | --- |
| IPFB | 7.20 | 7.65 | 294 | 167 | 117 | 26 | 33 |
| 24DFIB | 7.80 | 8.20 | 240 | 113 | 63 | 21 | 33 |
| IB | 8.20 | 8.70 | 204 | 77 | 204 | 18 | 1 |
| 4IMB | 9.90 | 10.40 | 218 | 91 | 65 | 18 | 30 |
| 3C4FIB | 10.80 | 11.20 | 256 | 129 | 109 | 20 | 31 |
| 1C2IB | 11.25 | 11.60 | 238 | 111 | 75 | 18 | 32 |
| 2I13DMB | 11.65 | 12.10 | 232 | 105 | 77 | 18 | 32 |
| 1B4IB | 12.25 | 12.65 | 282 | 155 | 157 | 20 | 18 |
| 24DCIB | 13.40 | 13.90 | 272 | 145 | 109 | 19 | 30 |
| 245TCIB | 15.60 | 16.10 | 308 | 181 | 179 | 21 | 32 |

**Chromatogram comparison**

In Fig. S 4 the chromatograms of the 50 ng/L standard measured with the three techniques can be seen. As expected, GC-EI-MS is the least selective technique, since not only AA derivatives can be seen. Saturation of several compounds (marked with red) and tailing can also be observed in the GC-NCI-MS chromatogram. This is in agreement with the upper limit of the linear ranges found for this technique, which were ≤ 50 ng/L for all compounds. If concentrations in this order of magnitude are of interest, doing split injections or diluting the samples would reduce and/or eliminate the tailing and detector saturation.

a)

1

2

3

4

5

6

7

8

9

10

b)

1

2

3

4

5

6

7

8

9

10

c)

1

2

3

4

5

6

7

8

9

10

Fig. S 4. Chromatogram comparison of the 50 ng/L level measured with a) GC-EI-MS, b) GC‑NCI‑MS, and c) GC-EI-MS/MS. The m/z shown are a) the quantifier ions reported in Table S 2, b) 127 and c) the transitions reported in Table S 1. The peaks correspond to: 1. IPFB, 2. 24DFIB, 3. IB, 4. 4IMB, 5. 3C4FIB, 6. 1C2IB, 7. 2I13DMB, 8. 1B4IB, 9. 24DCIB, 10. 245TCIB.

**Exemplary calibration curves**

Fig. S 5. Concentration curves obtained with each of the techniques studied, for some of the analytes with the biggest linear range for each technique. The linear equations and the regression coefficients can be found within each graph. The prediction bands were calculated according to DIN 32645[4].

**1B4IB with GC-EI-MS: problematic analyte**

The high background noise observed (Fig. S 6) hinders the proper identification of IB4IB in lower calibration levels (< 10 ng/L).

Fig. S 6. Chromatogram comparison of the m/z = 282, corresponding to the quantifier ion of 1B4IB, of the 5 ng/L (pink) and 50 ng/L (black) calibration standards, measured with GC‑EI‑MS.

**Detailed intra-day and inter-day repeatability results**

Intra-day repeatability values below 15 % were obtained for all analytes, concentration levels and measuring techniques with three exceptions, all in the lower concentration range: 1B4IB with GC‑EI-MS, IB with GC-EI-MS/MS and 245TCIB with the same instrument (see Table S 4). The concentration level tested for IB4IB (1 ng/L) was below the linear range of the analyte for that technique. In the case of GC‑EI-MS/MS, the concentration levels used were 10 pg/L, well below any previously reported limit (Table 3, main paper). In the case of 245TCIB, the low intra-day repeatability could be attributed to the fact that the concentration tested was the lowest concentration that could be detected.

Before the second repetition of the repeatability experiments with GC-EI-MS, real samples with relatively high concentrations were measured. This led to contaminated blanks for 24DFIB and IB, which had to be corrected for in the lower concentration level, and could account for the worse precision observed for those analytes. In the case of GC-EI-MS/MS, IB and 4IMB were found in all the blanks from the precision experiments and also had to be corrected for in the lower calibration level. In this case, that could be because the concentration range tested for these experiments is four orders of magnitude broad, and the fiber might need extra conditioning to avoid carryover for subsequent measurements at extremely low concentrations (10 pg/L). This can also be observed in the results, especially for IB. Furthermore, in one of the repetitions the syringe blank (where pure methanol was added, instead of a stock solution, after cleaning the syringe 10-20 times) appeared contaminated, which emphasizes the difficulty of working with such small concentrations and the importance of taking extra steps to make sure everything is clean.

Table S 4. Intra-day and inter-day repeatability (%) results obtained for the iodinated aromatic compounds. The concentration levels tested were: for GC-EI-MS, L (low) = 1 ng/L, M (medium) = 10 ng/L, H (high) = 100 ng/L; for GC-NCI‑MS, L = 0.1 ng/L, M = 1 ng/L, H = 10 ng/L; and for GC-EI-MS/MS, L = 0.01 ng/L, M-L = 0.1 ng/L, M-H = 1 ng/L, H = 10 ng/L. Results in bold are above 20 %. GC‑MS/MS results are normalized according to the SPME Mix intensities over time (described in SI).

| **Intra-day repeatability (%, n = 9)** | | | | | | | | | | | | | |
| --- | --- | --- | --- | --- | --- | --- | --- | --- | --- | --- | --- | --- | --- |
|  |  | **GC-EI-MS** | | |  | **GC-NCI-MS** | | |  | **GC-EI-MS/MS** | | | |
|  |  | L | M | H* |  | L | M | H |  | L | M-L | M-H | H |
| IPFB |  | 10* | 11 | 6.8 |  | 4.8 | 5.0* | 5.0 |  | 6.1* | 2.5 | 1.4* | 1.3 |
| 24DFIB |  | 11 | 10 | 4.6 |  | 2.4 | 5.0* | 5.3 |  | 13 | 2.5 | 2.2 | 1.8* |
| IB |  | 9.9 | 5.5* | 6.4 |  | 11 | 3.4* | 3.8 |  | 19 | 3.2 | 4.1 | 1.9 |
| 4IMB |  | 5.0* | 10 | 4.5 |  | 12 | 8.8* | 3.3* |  | 8* | 1.9* | 3.3 | 1.9 |
| 3C4FIB |  | 2.8* | 6.6 | 6.0 |  | 3.7 | 7.3 | 4.3 |  | 8.9 | 5.2* | 0.2* | 2.4* |
| 1C2IB |  | 7.5 | 9.3 | 5.1 |  | 2.9 | 4.8 | 3.3* |  | 12 | 2.0 | 3.0 | 2.3 |
| 2I13DMB |  | 6.9* | 5.5 | 5.5 |  | 2.9 | 3.3 | 2.1 |  | 7.7 | 3.2 | 2.5 | 1.3 |
| 1B4IB |  | - | 7.6* | 4.2 |  | 3.8 | 8.4 | 3.7 |  | 11* | 5.4 | 4.3 | 2.6 |
| 24DCIB |  | 3.8 | 5.7 | 5.2 |  | 3.0* | 3.4 | 4.0 |  | 11 | 4.6* | 3.2 | 2.4 |
| 245TCIB |  | 6.8 | 5.3 | 3.9 |  | 9.2* | 5.5 | 2.7 |  | **28** | 10* | 3.5 | 2.9 |

| **Inter-day repeatability (%, n = 3)** | | | | | | | | | | | | | |
| --- | --- | --- | --- | --- | --- | --- | --- | --- | --- | --- | --- | --- | --- |
|  |  | **GC-EI-MS** | | |  | **GC-NCI-MS** | | |  | **GC-EI-MS/MS** | | | |
|  |  | L | M | H* |  | L | M | H |  | L | M-L | M-H | H |
| IPFB |  | 1.8* | 4.0 | 9.1 |  | 12 | 20* | 20 |  | 8.9* | 12 | 13* | 12 |
| 24DFIB |  | 12 | 17 | 6.6 |  | 18 | 17* | **28** |  | **21** | 10 | 19 | 12* |
| IB |  | 16 | 8.9* | 6.2 |  | 14 | 16* | **21** |  | **25** | **22** | **27** | 18 |
| 4IMB |  | **46*** | **22** | 6.9 |  | 12 | **28*** | 19* |  | 6.0* | 20* | **30** | 18 |
| 3C4FIB |  | 8.0* | 3.3 | 4.7 |  | 13 | **26** | **25** |  | 13 | 20* | 15* | 16* |
| 1C2IB |  | **46** | **21** | 10 |  | 12 | **34** | **28*** |  | 4.0 | 7.2 | 19 | 12 |
| 2I13DMB |  | **25*** | 12 | 9.2 |  | 9.3 | **40** | **32** |  | 9.1 | 14 | 16 | 9.9 |
| 1B4IB |  | - | 19* | 11 |  | 3.7 | **27** | 19 |  | **24*** | 20 | **30** | **22** |
| 24DCIB |  | **31** | 13 | 6.9 |  | 16* | **34** | **31** |  | 8 | 13* | **21** | 16 |
| 245TCIB |  | **41** | **24** | 5.9 |  | 19* | **31** | **23** |  | **35*** | 20* | **22** | 18 |

*Outliers found with Dixon´s Q test (α = 0.05, Q_Critical_ = 0.436 and 0.941), not included in the calculations.

**Inter-day repeatability: repetitions over time**

Fig. S 7. Peak area over time at the same concentration level for each method studied. Repetitions 1-3, 4-6 and 7-9 were performed in consecutive days. All outliers are included.

**Internal standard-equivalent correction**

Ideally, one isotopically labelled internal standard per analyte of interest is used both during the validation of the method, and the analysis of real samples. However, sometimes they are not commercially available or their price is extremely high. In this study, an equivalent correction was used during the LOD and precision/recovery experiments, but instead of based on a specific internal standard, it was based on the overall response of all the analytes.

First, the peak areas obtained for each analyte were normalized based on their average (analyte correction factor), afterwards, these results were added for each sample (sample correction factor) and the average was calculated, and finally, each sample correction factor was normalized by the average (internal standard equivalent) and used for the corresponding sample. In Table S 5 an example calculation can be seen for two analytes and two samples, and Fig. S 8 shows the corrected results for GC-NCI-MS (non-corrected results can be seen in Fig. S 7).

Table S 5. Exemplary calculation of the internal standard-equivalent correction.

| Repetition |  | 1 | 2 | Average |
| --- | --- | --- | --- | --- |
| Peak area IPFB |  | 616126 | 650372 | 633249 |
| Peak area 24DFIB |  | 2888157 | 3129281 | 3008719 |
| IPFB correction factor |  | 0.97 | 1.03 | - |
| 24DFIB correction factor |  | 0.96 | 1.04 | - |
| Sample correction factor |  | 1.93 | 2.07 | 2 |
| Internal standard equivalent |  | 0.97 | 1.03 | - |

Fig. S 8. Peak area over time for 10 ng/L measured with GC‑NCI‑MS, after internal standard equivalent correction. Repetitions 1-3, 4-6 and 7-9 were performed in consecutive days. All outliers are included.

**Detailed recovery results**

The recoveries obtained for each of the analytes can be seen in Table S 6. Some analytes showed recoveries below 80 %. In the case of GC‑MS, 1IB4IB could not be seen in the low concentration level (1 ng/L) as discussed in the main paper (section “Linear range”), and shown in Fig. S 6. The lower recoveries obtained when not optimal calibration curves are used can be seen, for example, at the low concentration levels for 245TCIB measured with GC-MS, and 1B4IB measured with GC‑NCI‑MS and at the high concentration level for 24DFIB measured with GC‑NCI‑MS. The effect of the bigger tailing in the GC-MS/MS recovery results, as described in the main text, is more prominent for those analytes with a higher intensity than the rest, like 4IMB, 2I13DMB, and IB.

The GC-EI-MS results for the analytes 24DFIB and IB for the lower concentration levels show that the blank correction mentioned in the section “Detailed intra-day and inter-day repeatability results” had no adverse effect in the recoveries obtained and corroborating the need for such a correction. In the case of the GC-EI-MS/MS results, 4IMB might seem like it was over-corrected for, since the recovery obtained is only 81 %. However, this value is very similar in the other concentration levels, and, when compared with the other analytes within the same level, it is consistently in the lower range.

Table S 6. Recovery (%) results obtained for the iodinated aromatic compounds, including average and relative standard deviation (RSD). Results in bold are below 80 %. The concentration levels tested can be seen in Table S 4. GC‑EI-MS/MS results are normalized according to the SPME Mix intensities over time.

| **Recovery (%, n = 9)** | | | | | | | | | | | | | |
| --- | --- | --- | --- | --- | --- | --- | --- | --- | --- | --- | --- | --- | --- |
|  |  | **GC-EI-MS** | | |  | **GC-NCI-MS** | | |  | **GC-EI-MS/MS** | | | |
|  |  | L | M | H* |  | L | M | H |  | L | M-L | M-H | H |
| IPFB |  | 94 | 107 | 107 |  | 97 | 99* | 84 |  | 102 | 103 | 88 | **74** |
| 24DFIB |  | 94 | 106 | 100 |  | **73** | 85* | **79** |  | 90 | **78** | **76** | **69** |
| IB |  | 99 | 110* | 104 |  | 82 | 88* | 80 |  | 92 | 87 | **79** | **74** |
| 4IMB |  | 105 | 107 | 99 |  | 82 | 93 | 81 |  | 81 | 81 | 80 | **79** |
| 3C4FIB |  | 99* | 99 | 99 |  | 91 | 104 | 84 |  | 98 | 87 | 90 | 83 |
| 1C2IB |  | 111 | 107 | 99 |  | 82 | 90 | 80 |  | **66** | 80 | 85 | 80 |
| 2I13DMB |  | 98 | 102 | 100 |  | 80 | 85 | **74** |  | 80 | **68** | **75** | **75** |
| 1B4IB |  | - | 95 | 93 |  | **71** | 91 | 82 |  | 106 | 97 | 96 | 87 |
| 24DCIB |  | 108 | 101 | 95 |  | 84 | 92 | 80 |  | 101 | 98 | 93 | 83 |
| 245TCIB |  | 116 | 107 | 96 |  | 85 | 98 | 81 |  | 102 | 114 | 117 | 96 |
| Average |  | 102 | 104 | 96 |  | 83 | 94 | 80 |  | 92 | 89 | 88 | 80 |
| RSD |  | 8 | 4 | 4 |  | 8 | 6 | 3 |  | 13 | 14 | 12 | 8 |

*Outliers found with Dixon´s Q test (α = 0.05, Q_Critical_ = 0.436), not included in the calculations.

**High concentrations with GC-EI-MS/MS: tailing outside of measuring windows**

It is believed that the SPME fiber was not sufficiently conditioned (despite following the manufacturer´s instructions) when the first experiments, namely the calibration curve, were measured with GC‑EI-MS/MS. Therefore, a lower intensity can be observed for those experiments (Fig. S 9). Furthermore, because a narrow window was set for each analyte in the MS/MS parameters in order to increase the selectivity, when the intensities were higher and bigger tailing occurred, this was not always fully recorded (inset, Fig. S 9). This could lead to lower recoveries than expected, especially for those analytes with more tailing or narrower windows.

Fig. S 9. Chromatogram comparison of the 10 ng/L level measured with GC-EI-MS/MS during the calibration curve experiments (black) and the LOD, LOQ, and recovery experiments (exemplary from the first day, pink) (see Table S 3 for recorded precursor/product ions). In contrast to 24DCIB (right), 1B4IB (left) shows some tailing (inset, exemplary from the third day) that continues past the recorded time.

**Real samples concentrations**

The values shown in Table S 7 were used for the calculations of the average concentrations of Table 5 (main paper), with the exception of the values marked with *. These values were excluded for different reasons. In the case of 4IMB, we believe the slight differences in the columns used enabled the separation of isomers with GC-EI-MS, but not with the other techniques, as seen in the comparison with GC-EI-MS/MS in Fig. S 10.

Fig. S 10. Chromatograms corresponding to 4IMB in S4 samples, measured with GC-EI-MS (left) and GC-EI-MS/MS (right).

In the case of 3C4FIB and 245TCB, it is believed that the analytes of interest might have co-eluted with other iodinated analytes when measured with GC-NCI-MS. Because with the other two techniques only analytes with a defined m/z ratio are detected, co-eluting compounds with different molecular ions can be successfully avoided. GC-NCI-MS does not provide information regarding the molecular ions, and can therefore lead to higher concentrations. A possibility would be to combine GC-NCI-MS with one of the other techniques discussed here. Another alternative could be GCxGC-NCI-MS, since a significantly higher chromatographic resolution can be achieved, minimizing potential co-elutions.

Table S 7. Calculated concentrations in the urine samples from three NS = non-smoker and four S = smoker donors, in ng/L. The techniques are abbreviated: GC: GC-EI-MS; NCI: GC-NCI-MS; MSMS: GC-EI-MS/MS. n.d. = not detected, > CC = results above the upper calibration curve limit. Results not considered for the average calculations marked with *.

|  | NS1 | | | NS2 | | | NS3 | | | S1 | | | S2 | | | S3 | | | S4 | | |
| --- | --- | --- | --- | --- | --- | --- | --- | --- | --- | --- | --- | --- | --- | --- | --- | --- | --- | --- | --- | --- | --- |
|  | GC | NCI | MSMS | GC | NCI | MSMS | GC | NCI | MSMS | GC | NCI | MSMS | GC | NCI | MSMS | GC | NCI | MSMS | GC | NCI | MSMS |
| IPFB | n.d. | n.d. | n.d. | n.d. | n.d. | n.d. | n.d. | n.d. | n.d. | n.d. | n.d. | n.d. | n.d. | n.d. | n.d. | n.d. | n.d. | 0.07 | n.d. | n.d. | n.d. |
| 24 DFIB | n.d. | n.d. | 0.04 | n.d. | n.d. | 0.05 | n.d. | n.d. | 0.04 | n.d. | n.d. | 0.05 | n.d. | n.d. | 0.07 | n.d. | n.d. | 0.07 | n.d. | n.d. | 0.08 |
| IB | >CC | >CC | 823 | >CC | >CC | >CC | >CC | >CC | >CC | >CC | >CC | >CC | >CC | >CC | >CC | >CC | >CC | >CC | >CC | >CC | >CC |
| 4IMB | 14 | 47 | 5.0* | 68 | 60 | 7.1* | 11.3 | 39 | 1.6* | 89 | 67 | 4.1* | 237 | 110 | 19* | >CC | 130 | 54* | >CC | 145 | 79* |
| 3C4 FIB | 0.8 | 63* | 0.5 | 0.7 | 69* | 0.5 | 0.6 | 63* | 0.5 | 0.6 | 63* | 0.6 | 0.4 | 62* | 0.4 | 1.1 | 83* | 0.9 | 1.1 | 78* | 0.8 |
| 1C2IB | 13 | 16 | 10 | 19 | 15 | 18 | 12 | 14 | 14 | 22 | 20 | 24 | 18 | 17 | 21 | 34 | 34 | 34 | 28 | 30 | 28 |
| 2I13 DMB | 1.7 | 5 | 1.4 | 2.8 | 7 | 2.2 | 1.5 | 1.9 | 1.4 | 3.4 | 9 | 3 | 4.9 | 62 | 4.4 | 19 | 32 | 12 | 60 | 41 | 38 |
| 1B4IB | n.d. | n.d. | 2.5 | n.d. | n.d. | 6.3 | n.d. | n.d. | 6.2 | n.d. | n.d. | 15 | 103 | 173 | 129 | n.d. | n.d. | 15 | n.d. | n.d. | 17 |
| 24 DCIB | 0.3 | n.d. | 0.2 | 0.2 | n.d. | 0.2 | 0.4 | n.d. | 0.3 | 0.3 | n.d. | 0.3 | n.d. | n.d. | 0.4 | 0.5 | n.d. | 0.6 | 0.8 | n.d. | 0.9 |
| 245TCIB | n.d. | 0.5 | 0.5 | n.d. | 12* | 0.7 | n.d. | n.d. | 1.2 | n.d. | 5.0 | 0.7 | n.d. | 111* | 0.8 | n.d. | 9.3* | 0.8 | 0.2 | 168* | 0.8 |

**References**

1. Domínguez I, Arrebola FJ, Gavara R, Martínez Vidal JL, Frenich AG. Automated and simultaneous determination of priority substances and polychlorinated biphenyls in wastewater using headspace solid phase microextraction and high resolution mass spectrometry. Anal Chim Acta. 2018;1002:39-49. <https://doi.org/10.1016/j.aca.2017.11.056>.

2. de Perre C, Le Ménach K, Ibalot F, Parlanti E, Budzinski H. Development of solid-phase microextraction to study dissolved organic matter—Polycyclic aromatic hydrocarbon interactions in aquatic environment. Anal Chim Acta. 2014;807:51-60. <https://doi.org/10.1016/j.aca.2013.11.026>.

3. Bienfait B, Ertl P. JSME: a free molecule editor in JavaScript. Journal of Cheminformatics. 2013;5:24. <https://doi.org/10.1186/1758-2946-5-24>.

4. International Organization for Standardization, DIN EN ISO 32645:2008-11, Chemical analysis - Decision limit, detection limit and determination limit under repeatability conditions - Terms, methods, evaluation. (2008).
